# Dietary precursors and cardiovascular disease: A Mendelian randomization study

**DOI:** 10.3389/fcvm.2023.1061119

**Published:** 2023-02-09

**Authors:** Wangwei Jing, Shushi Huang, Pingping Xiang, Jiniu Huang, Hong Yu

**Affiliations:** ^1^Department of Cardiology, The Second Affiliated Hospital, School of Medicine, Zhejiang University, Hangzhou, China; ^2^Department of Cardiology, The Second Affiliated Hospital, Zhejiang University School of Medicine, Hangzhou, China

**Keywords:** dietary precursors, cardiovascular disease, Mendelian randomization (MR) analysis, choline, carnitine

## Abstract

**Background:**

The Dietary precursor has been identified as a contributor in the development of cardiovascular disease. However, it is inconsistent if dietary precursors could affect the process of cardiovascular disease.

**Methods:**

Here we performed Mendelian randomization (MR) analysis of the data from genome-wide association study of European ancestry to evaluate the independent effects of three dietary precursors on cardiovascular disease (CVD), myocardial infarction (MI), heart failure (HF), atrial fibrillation (AF), and valvular disease (VHD). Inverse variance weighting method was used for the MR estimation. Sensitivity was determined by MR-PRESSO analysis, weighted median analysis, MR-Egger analysis, and Leave-one-out analysis.

**Results:**

We found that elevated choline level had a causal relationship with VHD [odds ratio (OR) = 1.087, 95% confidence interval (CI), 1.003–1.178, *P* = 0.041] and MI (OR = 1.250, 95% CI, 1.041–1.501, *P* = 0.017) by single-variable MR analysis. Furthermore, elevated carnitine level was associated with MI (OR = 5.007, 95% CI, 1.693–14.808, *P* = 0.004) and HF (OR = 2.176, 95% CI, 1.252–3.780, *P* = 0.006) risk. In addition, elevated phosphatidylcholine level can increase the risk of MI (OR = 1.197, 95% CI, 1.026–1.397, *P* = 0.022).

**Conclusion:**

Our data show that choline increases VHD or MI risk, carnitine increases the risk of MI or HF, and phosphatidylcholine increases HF risk. These findings suggest the possibility that decrease in choline level in circulation may be able to reduce overall VHD or MI risk, reduce in carnitine level could be decrease MI and HF risks as well as decrease in phosphatidylcholine could reduce MI risk.

## 1. Introduction

With the development of economy and the sharp increase of aged population, cardiovascular disease becomes a major contributor to the loss of physical function, quality of life and longevity. Ischemic heart disease and other cardiovascular diseases (CVD) kill 17.5 million people worldwide each year (1), though the treatment of CVD has advanced. According to a large number of studies, many factors are involved with the initiation and progress of CVD, such as lipid accumulation (2, 3), inflammation (4–6), phosphate processing (7), biosynthesis and aggregation of extracellular vesicles (8, 9), cell senescence (10–12) and so on. However, there is still a lack of effective prevention of CVD development.

Traditionally, diet has been considered a major determinant of cardiovascular health. In fact, one of the seven cardiovascular health indicators proposed by the American Heart Association in 2010 (Life’s Simple 7) directly corresponds to a healthy diet (13). Choline is an essential nutrient from diet for humans throughout their life. Most individuals need to increase choline ingestion through their diet in order to prevent deficiency (14). Choline metabolism can be divided into four main pathways involved in the synthesis of acetylcholine, betaine, phospholipids and trimethylamine (TMA). Carnitine, a kind of amino acid, occurs only in nature as L-carnitine and is often used as a nutritional supplement which can also be converted into TMA with the help of intestinal flora (15). Phosphatidylcholine is not only the main component of cell membrane, but also necessary for cell division and growth (14, 16). All three nutrients can be digested in the gut into other substances, so here they are called dietary precursors (17). In this case, we wanted to explore the causal relationship between these dietary precursors and cardiovascular disease, such as total cardiovascular disease (CVD), myocardial infarction (MI), heart failure (HF), atrial fibrillation (AF) and valvular disease (VHD).

The Mendelian randomization (MR) technique is a novel method of assessing causal inference between risk factors and outcomes by using genetic variants as instrumental variables (IVs) (18). Genetic information is generally transmitted vertically from parents to their offspring, which is less susceptible to confounding and reverse causality and regarded as the nature’s randomized trial. MR studies using a solo-single nucleotide polymorphism (SNP) instrument found exposures associated with outcomes, which is called single-variable Mendelian randomization (SVMR) analysis.

Up to now, many independent SNPs have been identified as linked to choline, carnitine and phosphatidylcholine through genome-wide association studies (GWAS) (19, 20). However, the causal relationships between these dietary precursors and CVD from the current studies were contradictory and a point of view is that supplementation with dietary precursors improves prognosis during heart failure or MI (21–25). Another idea is that dietary precursors increase the risk of cardiovascular disease (26–28). In this research, we aimed to use the two-sample MR analysis of SVMR to determine the causal effect of above mentioned three dietary precursors on CVD.

## 2. Materials and methods

**FIGURE 1:**
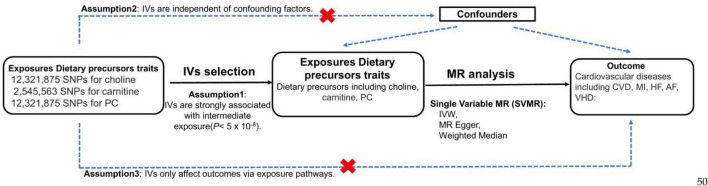
MR study flow diagram to determine the causal effect of three dietary precursors on five cardiovascular diseases. Dashed lines indicate potential pleiotropic or direct causal effects between variables that may violate MR assumptions. PC, phosphatidylcholine; IV, instrumental variable; IVW, multiplicative random effects inverse variance weighted; CVD, cardiovascular disease; MI, myocardial infarction; HF, heart failure; AF, atrial fibrillation; VHD, valvular heart disease.

This study conduct a SVMR analysis to investigate genetically association of three exposures (choline, phosphatidylcholine and carnitine) with five outcomes (CVD, MI, HF, AF, VHD) risk. An overview of the fundamentals, design, and process of our MR study is elucidated in Figure 1.

### Study design

This study followed the Strengthening the Reporting of Observational Studies in Epidemiology Using Mendelian Randomization (STROBE-MR) guide (29). To conduct the MR analysis, three assumptions must be met: (1) IVs are strongly associated with intermediate exposure (5*10^–8^), (2) IVs are independent of confounding factors, (3) IVs only affect outcomes via exposure pathways.

### Data source

We extracted the SNPs that were strongly associated with Choline (met-d-Cholines), carnitine (met-a-379) and phosphatidylcholines (met-d-Phosphatidylc) from the datasets archived in the GWAS database.^1^ For the same situation, we obtained summary-level data for the outcome events (CVD, MI, HF, AF, VHD) from the “finn-b-I9_CVD, finn-b-I9_MI, ebi-a-GCST009541, ebi-a-GCST006414, finn-b-I9_VHD” dataset. All SNPs were selected from European populations to eliminate demographic distribution bias and the GWAS data are exhibited in Table 1.

**TABLE 1:** Basic information about the GWAS database.

Exposure	GWAS-ID	PMID	Population	Number of SNPs	Sample size
Choline	met-d-Cholines	NA	European	12,321,875	114,999
Carnitine	met-a-379	24816252	European	2,545,563	7,797
PC	met-d-Phosphatidylc	NA	European	12,321,875	114,999
**Outcome**	**GWAS-ID**	**PMID**	**Population**	**Number of SNPs**	**Sample size**
CVD	finn-b-I9_CVD	NA	European	16,380,466	ncase: 111,108 ncontrol: 107,684
MI	finn-b-I9_MI	NA	European	16,380,433	ncase: 12,801 ncontrol: 187,840
HF	ebi-a-GCST009541	31919418	European	7,773,021	ncase: 47,309 ncontrol: 930,014
AF	ebi-a-GCST006414	30061737	European	33,519,037	ncase: 60,620 ncontrol: 970,216
VHD	finn-b-I9_VHD	NA	European	16,380,358	ncase: 38,209 ncontrol: 156,711

Information about exposures and outcome are sourced from the GWAS public database. PC, phosphatidylcholine; CVD, cardiovascular disease; MI, myocardial infarction; HF, heart failure; AF, atrial fibrillation; VHD, valvular heart disease; ncase, number of case; ncontrol, number of control.

In order to ensure that SNPs were strongly associated with each exposure, we extracted the SNPs at genome-wide significance level (*P*<5×10^–8^) from the GWAS datasets as their respective IVs. Afterward, trait-specific SNPs that were located at a distance of 1000 kb apart from each other were selected, and those in linkage disequilibrium (LD) (*r*^2^<0.001) were excluded. Then, we extracted the SNPs that were strongly associated with outcomes from the three dietary precursors associated SNPs at a correlation criterion of *r*^2^<0.01, respectively. Furthermore, we harmonized the data for exposures and outcomes by removing palindromic SNPs from the selected SNPs above.

For the SVMR analyses, we are using three methods (29): Inverse-variance weighted (IVW), MR-Egger and Weighted Median (WM), to assess the evidence of the causal effects of each TMA-related metabolites on VHD risk and disease outcomes, and to detect the sensitivity of the results to different patterns of violations of IV assumption (30, 31). The IVW method uses a meta-analysis approach to combine the Wald ratios of the causal effects of each SNP, relying on the assumption that all SNPs are valid IVs with no evidence of pleiotropy, so it was used as the major analysis. The MR-Egger and WM methods were used as secondary analyses to examine the robustness of the result (30). The MR-Egger method can detect and adjust for pleiotropy albeit with compromised power (30). The WM analysis can generate consistent estimate if at least 50% of the weight in the analysis comes from valid IVs (30). Effect estimates were reported in odds ratios (ORs) with corresponding 95% confidential intervals (CIs).

### Strength of SNPs in explaining phenotypic variation

First, we computed the proportion of phenotypic variation explained by each SNP (*R*^2^-value) in our IVW-SVMR model using the formula *R*^2^ = 2×(1 − MAF)×MAF×(β/SD)^2^ where SD is standard deviation followed by formula SD = SE×$\sqrt{\text{N}}$ and β is the coefficient for effect size (32), MAF is the minor allele frequency for each SNP, SE is the standard error for each SNP and N is the sample size. Then, a F-statistic was calculated to evaluate the total strength of our IVW-SVMR model for each lipid traits in explaining phenotypic variation using the formula *F* = (N − k − 1)/k×*R*^2^/(1 − *R*^2^) where N is the sample size, k is the total number of SNPs selected for MR analysis, and *R*^2^ is the total proportion of phenotypic variation explained by all the SNPs in IVW SVMR model (32). A F-statistic >10 suggests that SNPs in our IVW-SVMR model is a sufficiently strong instrument to explain phenotypic variation, while a F-statistic < 10 implies a weak instrument (32). And the results of F-statistic about three dietary precursors were shown in Supplementary Table 2. All F-statistics of dietary precursors were greater than 10.

## 3. Statistical analyses

Several statistical tests were performed to examine the existence of horizontal pleiotropy that violated the main MR assumptions. We calculated the effect estimate for each instrumental SNP on three exposures with the Wald estimator and assessed the possible measurement errors using the Delta method (33). The fixed-effects inverse variance-weighted (IVW) method was used as standard analysis to derive the final effect estimates. Heterogeneity among estimates of SNPs was measured by Cochran Q-derived p and the funnel plot (34). Sensitivity analyses included the multiplicative random-effects IVW (30), the weighted median (35), the MR-Egger regression method (36), and the MR-pleiotropy residual sum and outlier (MR-PRESSO) method (37). Where heterogeneity existed (Cochran Q-derived *P* < 0.05) (38), the multiplicative random-effects IVW method was adopted to avoid the bias of weak SNP-exposure associations (30). The weighted median method can provide valid estimates even when up to 50% of the information in the analysis comes from invalid IVs (35). The MR-Egger method provides more conservative causal estimates in the presence of pleiotropic variants and is less likely to generate inflated test statistics (36). The MR-PRESSO method was used to detect the presence of outliers that could bias the results (37). We applied the intercept test from MR-Egger to assess horizontal pleiotropy (39). In addition, we employed leave-one-out analyses to determine whether a single SNP drove the causal relationship. With this approach, we excluded one SNP in turn and then reevaluated the causal effect. Scatter plots depicting the associations were also provided. The P values in this study were 2-sided, and values < 0.05 were deemed as suggestive significance, whereas the highly reliable findings were those survivals with a Bonferroni-corrected threshold of 0.003 (0.05/15). All MR analyses were conducted using R software (version 4.1.0) with R packages including TwoSampleMR, MendelianRandomization, and MR-PRESSO.

## 4. Results

### SVMR analysis for the effects of three dietary precursors on five cardiovascular events

**FIGURE 2:**
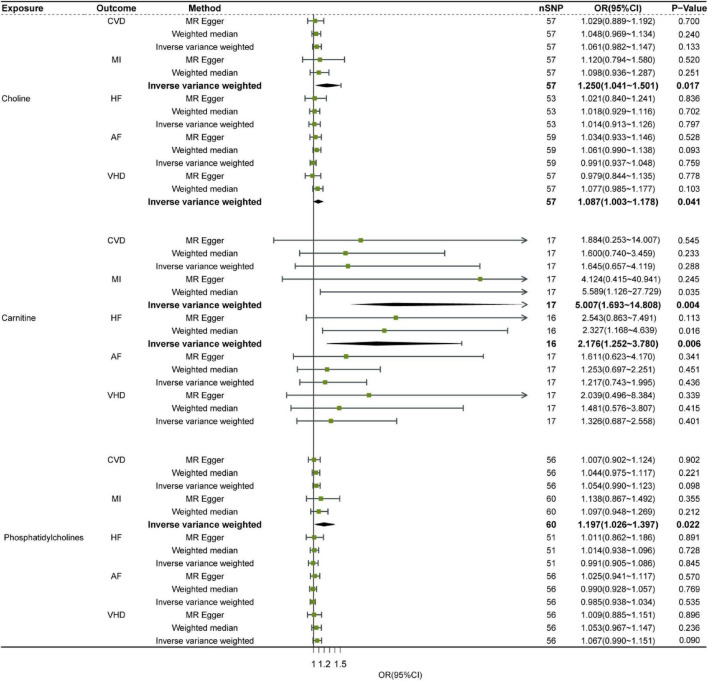
Relationship of Dietary precursors with five cardiovascular diseases by SVMR analysis. Sensitivity was determined by the listed methods. OR, odds ratio; CI, confidence interval; CVD, cardiovascular disease; MI, myocardial infarction; HF, heart failure; AF, atrial fibrillation; VHD, valvular heart disease.

Single nucleotide polymorphisms from the GWAS database listed on Table 1 were analyzed using SVMR. SNPs with LD distance >10,000 KB and MAF <0.01 as well as palindrome and multi-direction outlier SNPs were eliminated. Eventually, the numbers of SNP of three dietary precursors on five cardiovascular events were shown in Supplementary Table 1. The effects of genetic causality between three dietary precursors and five cardiovascular events were analyzed by the three MR methods (IVW, MR-Egger, WM) as shown in Figure 2. In addition, the effects (β values) of three dietary precursors on the risk of cardiovascular events were shown in the scatter plots (Supplementary Figure 1). The funnel plots of SVMR analysis for the effect of three dietary precursors on five outcomes are shown in four forest maps (Supplementary Figure 2) and four funnel plots (Supplementary Figure 3).

Although choline, carnitine and phosphatidylcholine showed significant heterogeneity in some outcomes, the most results remained consistent after MR-PRESSO analysis (Supplementary Table 1). This indicates that after data correction, the results show that increasing the level of choline and phosphatidylcholine can increase the risk of MI. No significant difference in pleiotropy was observed in the effect estimates of three exposures (Supplementary Table 1). Leave-one-out sensitivity analysis did not detect noticeable alterations in the effect estimation when any one SNP was removed (Supplementary Figure 4), suggesting robust results in our SVMR analysis.

In summary, out of three dietary precursors only choline was genetically associated with VHD based on IVW SVMR method, indicating that increased choline level had significantly higher risk of VHD (Figure 2). All three dietary precursors are associated with MI, meaning that increasing levels of any of them increases the risk of MI (Figure 2). And carnitine was also associated with odds of HF, this suggested that increasing the level of Carnitine increases the risk of HF (Figure 2).

## 5. Discussion

In this study, we found a causal relationship between high choline level and the risk of VHD by SVMR analyses based on the IVW SVMR method, while no causal relationship between the other precursors (phosphatidylcholine and carnitine) and VHD susceptibility (Figure 2). In addition, we found that high choline, carnitine, and phosphatidylcholine can all increase risk of MI also based on the IVW SVMR (Figure 2). Moreover, high carnitine is associated with high risk of HF based on the IVW SVMR (Figure 2).

Our conclusions from SVMR analysis are robust and reliable because the SVMR analysis with IVW method was confirmed with the analysis of MR-PRESSO and Heterogeneity (Supplementary Table 1). And the result of choline with VHD was in accordance with the conclusion from the previous observational studies via imaging modalities and histopathological examinations, in which it was concluded that choline was significantly associated with the presence and severity of calcific aortic stenosis (CAS) (40). In addition, our results suggested that levels of choline and phosphatidylcholine are associated with the risk of MI which was in line with some observational studies (41–44).

The result of carnitine with MI and HF from SVMR analysis indicated that increase level of carnitine can cause MI and HF which was consistent with some observational studies. Youngja H. Park’s research found that carnitine was significantly elevated in AMI risk sera (45). Furthermore, Sinha A found carnitine is associated with atherosclerotic risk and myocardial infarction in HIV -Infected adults (46) and animal experiments have found dietary L-carnitine promotes microbiota dependent atherosclerosis (47). But in some studies, carnitine administration to patients who had experienced on MI or HF is associated with a marked reduction in overall mortality, as confirmed by meta-analysis (22, 48). This may be attributed to the limitations of sample size and differences in inclusion criteria of meta-analysis, while the MR analysis happened to avoid these shortcomings.

### 5.1. Clinical importance

Our results from SVMR have potential implications for public health. First, our findings provide new information for understanding the causal influence of choline on the pathogenesis of VHD or MI, carnitine on the pathogenesis of MI or HF and phosphatidylcholine on the pathogenesis of MI. Second, our findings underscore the importance of screening high-risk VHD subjects in populations with elevated choline level, high-risk MI subjects in populations with elevated choline level, carnitine level or phosphatidylcholine. Furthermore, our results also shown importance of screening high-risk HF subjects in populations with elevated carnitine level. In addition, from a preventive perspective, our SVMR findings suggest a promising way to reduce the risk of cardiovascular diseases by adjusting the diet to minimizing the intake of foods.

### 5.2. Advantages

The current study has several advantages. To the best of our knowledge, our study is the first MR study to use large-scale GWAS data to focus on genetic causality between a range of dietary precursors and cardiovascular diseases risk. Our study with SVMR analysis is superior to the previous observational studies because we had large sample sizes and SNPs and identified a novel conclusion that the increased choline level was the primary causal factor for VHD and MI events, increased carnitine level was the dominant reason for MI and HF events as well as increased phosphatidylcholine level was the critical factor for MI event.

## 6. Limitations

We admit to several discomforts in our study. First, there are other risk factors, such as rheumatism and body mass index, that can contribute to exposure. However, our MR study was designed only to investigate the genetic causal effect of dietary precursors on the risk of five cardiovascular diseases. Anthropometry and rheumatic immune diseases were beyond the scope of our study. Second, our findings are based only on the European population and are not necessarily applicable to other populations. Due to the absence of GWAS database of other dietary precursors, we were unable to conduct a comprehensive analysis of dietary precursors about the risk of five cardiovascular diseases. Therefore, our conclusion should be used with caution. Thirdly, we cannot figure out exactly how choline causes VHD and MI, how carnitine causes MI and HF as well as how phosphatidylcholine causes MI. Experiments, and more observational studies are needed to determine whether these precursors play a key role in the progression of VHD, MI or HF and which specific mechanisms of precursors in the pathogenesis of cardiovascular diseases. Finally, concerns about the violation of some IV assumptions in nutritional research have been raised because nutrition is correlated with numerous other lifestyle and environmental factors *(49)*. In the current study, diet quality that might index overall nutritional status. However, residual confounding by other nuisance variables, especially those with a similar time trend (e.g., specific nutrients used to replace the choline, carnitine and phosphatidylcholine) that may attenuate the observed relations, could not be ruled out.

## 7. Future directions

The results of the study highlight genetic causal effect of dietary precursors on the risk of five cardiovascular diseases to use a single-variable Mendelian randomization. For the existing research results, we make the following outlook. Firstly, based on the analysis of the results of this study from the GWAS database, there must be possible biases caused by the data itself. Therefore, in the future, we may pay more attention to and elaborate the common interfering factors between exposures (dietary precursors) and outcomes (cardiovascular diseases) and explore the relationships between more different dietary precursors and cardiovascular diseases. Furthermore, the same study needs to be analyzed again in other populations to make sure the findings are universal. Secondly, as for the research results of this study, we need to conduct further basic research to verify again and explore the possible mechanism. Finally, because diet is linked to many other lifestyle and environmental factors, concerns have been raised about the violation of some IV assumptions in nutrition studies. Regarding this point, we will upgrade and improve the research methods through more detailed classification of the population and more precise inclusion criteria, hoping to find a way to reduce or eliminate the interference.

## 8. Conclusion

Our SVMR provide genetic evidence that elevated choline level mainly explains the causal effect on the risk of developing MI or VHD, elevated carnitine can increase the risk of MI or HF and elevated phosphatidylcholine can increase the risk of MI.

## Data availability statement

The original contributions presented in this study are included in the article/Supplementary material, further inquiries can be directed to the corresponding author.

## Ethics statement

The studies involving human participants were reviewed and approved by the UK Biobank. The patients/participants provided their written informed consent to participate in this study.

## Author contributions

WJ and HY: conceptualization. WJ: methodology, software, investigation, resources, visualization, writing—original draft preparation, visualization, and project administration. WJ, PX, and SH: validation. JH: formal analysis. PX: data curation and writing—review and editing. SH: supervision. HY: funding acquisition. All authors have read and agreed to the published version of the manuscript.

## Footnotes

1
https://gwas.mrcieu.ac.uk/
