# Supplementary material for: Dietary precursors and cardiovascular disease: A Mendelian randomization study
Source: Front Cardiovasc Med. 2023 Feb 9;10:1061119. doi: 10.3389/fcvm.2023.1061119 (PMC9947469; doi:10.3389/fcvm.2023.1061119)
Supplement: Supplementary file 1 [file Data_Sheet_1.PDF]

Supplement Table 1. Characteristic results of dietary precursors by SVMR analysis

Table 1-1. Characteristic results of choline by SVMR analysis

**Outcome**	**nSNP**	**Heterogeneity**		**Pleiotropy**		**MR-PRESSO**	
		Q-statistic	P-value	SE	P-value	OR (95%CI)	P-value
CVD	57	156.6893	<0.001	0.0041	0.6318	1.0485 0.9901~1.1069	0.1176
MI	57	212.1111	<0.001	0.0096	0.4624	1.2203 1.0843~1.3562	0.0060
HF	53	243.0098	<0.001	0.0056	0.9340	1.0348 0.9636~1.1059	0.3508
AF	59	109.5044	<0.001	00029	0.3436	NA	NA
VHD	57	118.5896	<0.001	0.0041	0.1060	1.0808 1.0083~1.1533	0.0402

Table 1-2. Characteristic results of carnitine by SVMR analysis

**Outcome**	**nSNP**	**Heterogeneity**		**Pleiotropy**		**MR-PRESSO**	
		Q-statistic	P-value	SE	P-value	OR (95%CI)	P-value
CVD	17	46.9677	<0.001	0.0073	0.8824	1.3360 0.7665~1.9055	0.3370
MI	17	10.5372	0.8371	0.0083	0.8534	NA	NA
HF	16	17.1835	0.3080	0.0043	0.7440	NA	NA
AF	17	21.4130	0.1632	0.0037	0.5060	NA	NA
VHD	17	17.1242	0.3776	0.0052	0.5081	NA	NA

Table 1-3. Characteristic results of phosphatidylcholines by SVMR analysis

Outcome	nSNP	Heterogeneity		Pleiotropy		MR-PRESSO	
		Q-statistic	P-value	SE	P-value	OR (95%CI)	P-value
CVD	56	111.2240	<0.001	0.0033	0.3191	1.0438 0.9883~1.0993	0.1356
MI	56	164.4221	<0.001	0.0081	0.6561	1.1874 1.0534~1.3216	0.0150
HF	51	190.2368	<0.001	0.0048	0.7609	0.9952 0.9280~1.0625	0.8890
AF	56	87.0782	0.0038	0.0026	0.2654	0.9882 0.9429~1.0334	0.6094
VHD	56	113.6591	<0.001	0.0039	0.3113	1.0618 0.9943~1.1292	0.0872

SVMR results of choline with cardiovascular disease. Q: Cochran's Q statistic ; OR: odds ratio; CI: confidence interval; nSNP: number of single nucleotide polymorphism; NA: the calculation result cannot be obtained due to the shortage of SNP.

Supplement Table 2. F-statistic of dietary precursors

Table 2-1. F-statistic of choline

	id.exposure	SNP	se.exposur	beta.expos	pos.exposure	eaf.exposu	N	SD	R2	F OF ONE	F-statistic
1	met-d-Cholines	rs1002687	0.004057	0.072981	62963737	0.644748	114999	1.375793	0.001289	2.317921	41.13472
2	met-d-Cholines	rs3768321	0.004896	-0.02815	40035928	0.196522	114999	1.660148	9.08E-05	0.163036	
3	met-d-Cholines	rs660240	0.004727	0.031259	109817838	0.78472	114999	1.603061	0.000128	0.230741	
4	met-d-Cholines	rs2642438	0.004254	0.031832	220970028	0.703815	114999	1.442517	0.000203	0.364667	
5	met-d-Cholines	rs496654	0.003885	0.022248	234851165	0.517239	114999	1.317618	0.000142	0.255738	
6	met-d-Cholines	rs7551124	0.005863	0.035204	23785760	0.875389	114999	1.988072	6.84E-05	0.122855	
7	met-d-Cholines	rs4846921	0.003985	0.025827	230304352	0.613253	114999	1.35137	0.000173	0.311195	
8	met-d-Cholines	rs1260326	0.003969	-0.06394	27730940	0.60401	114999	1.34611	0.001079	1.940221	
9	met-d-Cholines	rs672889	0.00559	0.067173	21319016	0.860091	114999	1.895738	0.000302	0.542815	
10	met-d-Cholines	rs1189535	0.003898	-0.03472	20367135	0.475398	114999	1.321874	0.000344	0.618351	
11	met-d-Cholines	rs3770586	0.003902	-0.02229	169828995	0.48397	114999	1.323088	0.000142	0.254698	
12	met-d-Cholines	rs4860948	0.004532	0.032407	69340991	0.244353	114999	1.536995	0.000164	0.294881	
13	met-d-Cholines	rs1310732	0.007405	-0.05009	103188709	0.074275	114999	2.511021	5.47E-05	0.098275	
14	met-d-Cholines	rs7707394	0.004047	0.022698	74472939	0.357106	114999	1.372378	0.000126	0.225579	
15	met-d-Cholines	rs5744672	0.003992	0.03213	74877803	0.384185	114999	1.353676	0.000267	0.478841	
16	met-d-Cholines	rs6882345	0.004024	0.02915	156397673	0.632863	114999	1.364728	0.000212	0.380822	
17	met-d-Cholines	rs7983416	0.010734	-0.06315	34889423	0.033987	114999	3.640162	1.98E-05	0.03549	
18	met-d-Cholines	rs1131285	0.00406	0.032702	31323953	0.648355	114999	1.376732	0.000257	0.462138	
19	met-d-Cholines	rs1866962	0.016388	-0.11706	161111700	0.014358	114999	5.557417	1.26E-05	0.022551	
20	met-d-Cholines	rs1401450	0.005787	0.031398	32597435	0.135289	114999	1.962506	5.99E-05	0.107554	
21	met-d-Cholines	rs1065344	0.004378	0.02554	73016541	0.715017	114999	1.484595	0.000121	0.21662	
22	met-d-Cholines	rs1057558	0.004689	-0.03307	1062366	0.220332	114999	1.590093	0.000149	0.266927	
23	met-d-Cholines	rs1992442	0.004407	0.032985	19853389	0.265042	114999	1.494334	0.00019	0.340962	
24	met-d-Cholines	rs2737214	0.003943	0.024119	116624987	0.558538	114999	1.337209	0.00016	0.288168	
25	met-d-Cholines	rs9987289	0.006758	0.092483	9183358	0.909151	114999	2.291831	0.000269	0.483198	
26	met-d-Cholines	rs1128756	0.004033	-0.0529	126506694	0.392346	114999	1.3676	0.000713	1.281902	
27	met-d-Cholines	rs2066714	0.005773	0.032646	107586753	0.128936	114999	1.957799	6.25E-05	0.112171	
28	met-d-Cholines	rs2740488	0.00441	-0.04836	107661742	0.265321	114999	1.495524	0.000408	0.732397	
29	met-d-Cholines	rs4008004	0.004695	0.031948	15300968	0.221842	114999	1.59225	0.000139	0.249657	
30	met-d-Cholines	rs1178960	0.006251	0.05369	107647019	0.108831	114999	2.119757	0.000124	0.223499	
31	met-d-Cholines	rs635634	0.005035	0.036102	136155000	0.183554	114999	1.707312	0.000134	0.24071	
32	met-d-Cholines	rs1480636	0.004666	-0.0324	45998984	0.763414	114999	1.58227	0.000152	0.272118	
33	met-d-Cholines	rs2792735	0.004326	-0.03567	113921825	0.720377	114999	1.467144	0.000238	0.427799	
34	met-d-Cholines	rs1174882	0.006018	-0.03837	5261832	0.131681	114999	2.040787	8.08E-05	0.145202	
35	met-d-Cholines	rs102275	0.004061	-0.03804	61557803	0.349755	114999	1.377292	0.000347	0.623404	
36	met-d-Cholines	rs5603082	0.00415	-0.03367	47397353	0.323119	114999	1.407348	0.00025	0.449872	
37	met-d-Cholines	rs7299761	0.006685	-0.06646	75474195	0.094064	114999	2.267092	0.000146	0.263032	
38	met-d-Cholines	rs525028	0.004293	-0.06665	116705516	0.708543	114999	1.455912	0.000866	1.555713	
39	met-d-Cholines	rs6606717	0.003881	0.028037	109873227	0.52317	114999	1.315963	0.000226	0.406795	
40	met-d-Cholines	rs1769673	0.003926	-0.02403	112486818	0.430221	114999	1.331508	0.00016	0.286836	
41	met-d-Cholines	rs7139079	0.003963	-0.03182	121415293	0.592501	114999	1.34376	0.000271	0.486257	
42	met-d-Cholines	rs3741521	0.004207	-0.02858	125264293	0.669712	114999	1.426559	0.000178	0.318857	
43	met-d-Cholines	rs1287821	0.004151	0.023112	24826201	0.670136	114999	1.407636	0.000119	0.214065	
44	met-d-Cholines	rs3466361	0.005752	0.042181	58569330	0.137654	114999	1.95061	0.000111	0.199393	
45	met-d-Cholines	rs261290	0.004089	-0.14661	58678720	0.654653	114999	1.386787	0.005054	9.121398	
46	met-d-Cholines	rs633695	0.004281	0.108287	58725839	0.292348	114999	1.451877	0.002302	4.142974	
47	met-d-Cholines	rs7591153	0.011454	-0.08176	57049137	0.032871	114999	3.884359	2.82E-05	0.050586	
48	met-d-Cholines	rs4986970	0.010762	-0.06314	67976320	0.033926	114999	3.649658	1.96E-05	0.035234	
49	met-d-Cholines	rs3764261	0.004147	0.098519	56993324	0.324455	114999	1.406357	0.002151	3.871637	
50	met-d-Cholines	rs7283656	0.011123	-0.08628	41926126	0.031483	114999	3.771841	3.19E-05	0.057311	
51	met-d-Cholines	rs2071379	0.003954	-0.02452	26695832	0.596255	114999	1.340986	0.000161	0.28909	
52	met-d-Cholines	rs7796034	0.01696	0.313193	47109955	0.013239	114999	5.751289	7.75E-05	0.139153	
53	met-d-Cholines	rs1176875	0.018471	0.115223	47147524	0.012011	114999	6.26376	8.03E-06	0.014423	
54	met-d-Cholines	rs9304381	0.005041	0.084939	47158234	0.818434	114999	1.709492	0.000734	1.318598	
55	met-d-Cholines	rs5854292	0.007407	-0.08844	19379549	0.074383	114999	2.511761	0.000171	0.306613	
56	met-d-Cholines	rs1421589	0.006086	-0.06161	11190534	0.116705	114999	2.06402	0.000184	0.329994	
57	met-d-Cholines	rs8103923	0.005818	-0.02949	46402383	0.129369	114999	1.972937	5.03E-05	0.090398	
58	met-d-Cholines	rs602662	0.003897	0.025674	49206985	0.534418	114999	1.321494	0.000188	0.337379	
59	met-d-Cholines	rs1065853	0.007165	-0.12628	45413233	0.080578	114999	2.429881	0.0004	0.718957	
60	met-d-Cholines	rs7223511	0.004512	-0.04143	45444524	0.705067	114999	1.530141	0.000305	0.547648	
61	met-d-Cholines	rs1769903	0.010748	-0.11681	11330942	0.033705	114999	3.644808	6.69E-05	0.120147	
62	met-d-Cholines	rs1883711	0.011414	0.061957	39179822	0.031191	114999	3.870625	1.55E-05	0.027809	
63	met-d-Cholines	rs1800961	0.011345	-0.09532	43042364	0.030192	114999	3.847226	3.6E-05	0.064564	
64	met-d-Cholines	rs5754102	0.00508	-0.03616	21916272	0.183245	114999	1.722535	0.000132	0.236883	

Table 2-2. F-statistic of Carnitine

	SNP	id.exposur	pval.expos	beta.expos	se.exposur	pos.exposur	eaf.exposu	N	SD	R2	F OF ONE SNP	F-statistic
1	rs1466788	met-a-37	3.05E-16	0.0074	9.00E-04	110618730	0.5932	7797	7.95E-02	4.18E-03	1.92E+00	24.03579738
2	rs735315	met-a-37	1.87E-09	-0.0054	9.00E-04	51331157	0.5057	7797	7.95E-02	2.31E-03	1.06E+00	
3	rs2279014	met-a-37	4.86E-08	-0.005	9.00E-04	219261176	0.367	7797	7.95E-02	1.84E-03	8.43E-01	
4	rs9842133	met-a-37	4.20E-12	-0.0064	9.00E-04	179664102	0.3351	7797	7.95E-02	2.89E-03	1.33E+00	
5	rs4860022	met-a-37	9.60E-10	-0.0055	9.00E-04	167693875	0.3362	7797	7.95E-02	2.14E-03	9.80E-01	
6	rs419291	met-a-37	3.10E-18	-0.0079	9.00E-04	131633355	0.6333	7797	7.95E-02	4.59E-03	2.11E+00	
7	rs1318251	met-a-37	3.36E-08	0.005	9.00E-04	78573790	0.6194	7797	7.95E-02	1.87E-03	8.56E-01	
8	rs6862024	met-a-37	8.99E-10	-0.0055	9.00E-04	150428871	0.3691	7797	7.95E-02	2.23E-03	1.02E+00	
9	rs2396004	met-a-37	4.31E-08	-0.0049	9.00E-04	43355851	0.5611	7797	7.95E-02	1.87E-03	8.58E-01	
10	rs1082158	met-a-37	1.28E-21	-0.0086	9.00E-04	61516587	0.4124	7797	7.95E-02	5.68E-03	2.61E+00	
11	rs1235619	met-a-37	3.69E-63	-0.0274	0.0016	61413353	0.1638	7797	1.41E-01	1.03E-02	4.76E+00	
12	rs1118362	met-a-37	3.00E-08	-0.005	9.00E-04	47212370	0.5324	7797	7.95E-02	1.97E-03	9.04E-01	
13	rs1162095	met-a-37	1.63E-08	0.0051	9.00E-04	65491255	0.5233	7797	7.95E-02	2.05E-03	9.42E-01	
14	rs1162097	met-a-37	2.35E-08	0.0051	9.00E-04	96018052	0.3674	7797	7.95E-02	1.91E-03	8.78E-01	
15	rs2114713	met-a-37	2.67E-08	0.005	9.00E-04	80528373	0.42	7797	7.95E-02	1.93E-03	8.84E-01	
16	rs3736438	met-a-37	3.19E-10	0.0057	9.00E-04	45486651	0.6111	7797	7.95E-02	2.45E-03	1.12E+00	
17	rs1270939	met-a-37	6.41E-09	-0.0053	9.00E-04	55292115	0.3714	7797	7.95E-02	2.08E-03	9.52E-01	

Table 2-3. F-statistic of Phosphatidylcholine

	exposure	beta.expos	se.exposur	pos.expos	pval.expos	chr.exposu	id.exposur	SNP	eaf.exposu	N	SD	R2	F OF ONE	F-statistic
1	Phosphati	0.028366	0.00397	2.3E+08	3.30E-13	1	met-d-Ph	rs1321257	0.613244	114999	1.346412	0.000211	0.396788	46.30915
2	Phosphati	0.03396	0.00584	23785760	1.40E-09	1	met-d-Ph	rs7551124	0.875389	114999	1.9805	6.41E-05	0.120873	
3	Phosphati	0.078719	0.004042	62963737	2.00E-87	1	met-d-Ph	rs1002687	0.644748	114999	1.37055	0.001511	2.851735	
4	Phosphati	0.030407	0.004238	2.21E+08	3.20E-14	1	met-d-Ph	rs2642438	0.703815	114999	1.437024	0.000187	0.351786	
5	Phosphati	-0.06469	0.003955	27730940	6.40E-61	2	met-d-Ph	rs1260326	0.60401	114999	1.341057	0.001113	2.099945	
6	Phosphati	-0.03656	0.003883	20367135	1.30E-21	2	met-d-Ph	rs1189535	0.475398	114999	1.316913	0.000385	0.724807	
7	Phosphati	0.023053	0.003915	1.7E+08	1.50E-09	2	met-d-Ph	rs2389602	0.448608	114999	1.327622	0.000149	0.281098	
8	Phosphati	-0.0667	0.005684	21271707	8.20E-33	2	met-d-Ph	rs3472231	0.136132	114999	1.927665	0.000282	0.530788	
9	Phosphati	-0.04874	0.007377	1.03E+08	6.70E-12	4	met-d-Ph	rs1310732	0.074275	114999	2.501716	5.22E-05	0.098345	
10	Phosphati	0.034842	0.004516	69340991	2.50E-15	4	met-d-Ph	rs4860948	0.244353	114999	1.531298	0.000191	0.360298	
11	Phosphati	0.027979	0.00401	1.56E+08	2.40E-12	5	met-d-Ph	rs6882345	0.632863	114999	1.359777	0.000197	0.370766	
12	Phosphati	0.033764	0.004758	74925162	1.40E-12	5	met-d-Ph	rs9293656	0.210218	114999	1.613357	0.000145	0.274056	
13	Phosphati	0.031238	0.004045	31323953	1.80E-12	6	met-d-Ph	rs1131285	0.648355	114999	1.371707	0.000236	0.445678	
14	Phosphati	-0.10795	0.016328	1.61E+08	5.50E-12	6	met-d-Ph	rs1866962	0.014358	114999	5.537138	1.08E-05	0.020269	
15	Phosphati	-0.06391	0.010695	34889423	9.00E-10	6	met-d-Ph	rs7983416	0.033987	114999	3.626869	2.04E-05	0.038424	
16	Phosphati	-0.03586	0.004698	1090505	5.90E-15	7	met-d-Ph	rs1270171	0.216755	114999	1.593173	0.000172	0.324215	
17	Phosphati	0.029076	0.004361	73016541	3.00E-11	7	met-d-Ph	rs1065344	0.715017	114999	1.479033	0.000157	0.296801	
18	Phosphati	-0.05204	0.004018	1.27E+08	6.80E-39	8	met-d-Ph	rs1128756	0.392346	114999	1.362517	0.000696	1.311471	
19	Phosphati	0.030744	0.004388	19855600	8.60E-12	8	met-d-Ph	rs1741116	0.265213	114999	1.488057	0.000166	0.313526	
20	Phosphati	0.025079	0.004326	1.44E+08	5.00E-09	8	met-d-Ph	rs5696066	0.277751	114999	1.467059	0.000117	0.220944	
21	Phosphati	0.085011	0.006733	9183358	8.60E-37	8	met-d-Ph	rs9987289	0.909151	114999	2.283309	0.000229	0.431556	
22	Phosphati	0.025802	0.003929	1.17E+08	5.10E-11	8	met-d-Ph	rs2737214	0.558538	114999	1.332234	0.000185	0.348609	
23	Phosphati	0.034104	0.005045	15305378	2.20E-12	9	met-d-Ph	rs581080	0.820407	114999	1.710822	0.000117	0.220669	
24	Phosphati	0.030712	0.005752	1.08E+08	1.80E-08	9	met-d-Ph	rs2066714	0.128936	114999	1.95064	5.57E-05	0.104923	
25	Phosphati	0.053646	0.006228	1.08E+08	8.90E-18	9	met-d-Ph	rs1178960	0.108831	114999	2.112005	0.000125	0.235838	
26	Phosphati	0.023497	0.004224	15303822	1.00E-08	9	met-d-Ph	rs1043572	0.303277	114999	1.432269	0.000114	0.214333	
27	Phosphati	-0.04801	0.004394	1.08E+08	1.20E-28	9	met-d-Ph	rs2740488	0.265321	114999	1.490058	0.000405	0.762805	
28	Phosphati	0.031529	0.005016	1.36E+08	4.40E-11	9	met-d-Ph	rs635634	0.183554	114999	1.701069	0.000103	0.194026	
29	Phosphati	-0.03592	0.00431	1.14E+08	3.80E-17	10	met-d-Ph	rs2792735	0.720377	114999	1.461718	0.000243	0.458535	
30	Phosphati	-0.03972	0.005996	5261832	1.30E-11	10	met-d-Ph	rs1174882	0.131681	114999	2.033235	8.73E-05	0.164416	
31	Phosphati	-0.03338	0.004649	45998984	2.70E-13	10	met-d-Ph	rs1480636	0.763414	114999	1.576417	0.000162	0.305214	
32	Phosphati	-0.07343	0.004277	1.17E+08	5.80E-69	11	met-d-Ph	rs525028	0.708543	114999	1.450541	0.001058	1.996521	
33	Phosphati	-0.06706	0.006661	75474195	2.30E-23	11	met-d-Ph	rs7299761	0.094064	114999	2.258726	0.00015	0.283107	
34	Phosphati	-0.03177	0.004056	47527052	2.10E-15	11	met-d-Ph	rs1083872	0.368821	114999	1.375596	0.000248	0.468152	
35	Phosphati	-0.03406	0.005812	61591636	1.10E-09	11	met-d-Ph	rs174565	0.127746	114999	1.970859	6.66E-05	0.125426	
36	Phosphati	0.032284	0.004311	1.25E+08	8.30E-14	12	met-d-Ph	rs1077311	0.720441	114999	1.461952	0.000196	0.370193	
37	Phosphati	-0.02614	0.004284	1.25E+08	1.90E-10	12	met-d-Ph	rs838886	0.696986	114999	1.452657	0.000137	0.257704	
38	Phosphati	-0.02102	0.003902	1.04E+08	2.40E-08	12	met-d-Ph	rs2292996	0.474903	114999	1.323091	0.000126	0.237214	
39	Phosphati	0.029267	0.003866	1.1E+08	4.40E-15	12	met-d-Ph	rs6606717	0.52317	114999	1.311076	0.000249	0.468558	
40	Phosphati	-0.03502	0.004007	1.21E+08	1.20E-18	12	met-d-Ph	rs7979473	0.612971	114999	1.358793	0.000315	0.593894	
41	Phosphati	-0.16448	0.004072	58678720	1.00E-200	15	met-d-Ph	rs261290	0.654653	114999	1.381039	0.006413	12.16211	
42	Phosphati	-0.16012	0.00554	58737341	1.50E-186	15	met-d-Ph	rs473224	0.853925	114999	1.878663	0.001812	3.420956	
43	Phosphati	-0.07548	0.004769	58577163	4.30E-59	15	met-d-Ph	rs1016264	0.210115	114999	1.617273	0.000723	1.363132	
44	Phosphati	0.102238	0.004131	56993324	1.70E-140	16	met-d-Ph	rs3764261	0.324455	114999	1.401003	0.002334	4.408902	
45	Phosphati	-0.0873	0.011411	57049137	6.00E-16	16	met-d-Ph	rs7591153	0.032871	114999	3.869574	3.24E-05	0.060973	
46	Phosphati	-0.03533	0.004911	72079657	2.30E-13	16	met-d-Ph	rs7730355	0.19269	114999	1.665479	0.00014	0.263896	
47	Phosphati	-0.0268	0.00394	26695832	7.20E-11	17	met-d-Ph	rs2071379	0.596255	114999	1.336066	0.000194	0.365017	
48	Phosphati	-0.08817	0.011082	41926126	5.20E-16	17	met-d-Ph	rs7283656	0.031483	114999	3.758005	3.36E-05	0.063258	
49	Phosphati	0.127471	0.018402	47147524	3.50E-12	18	met-d-Ph	rs1176875	0.012011	114999	6.240293	9.9E-06	0.01866	
50	Phosphati	0.328841	0.016896	47109955	1.00E-85	18	met-d-Ph	rs7796034	0.013239	114999	5.729755	8.61E-05	0.162168	
51	Phosphati	0.090752	0.005022	47158234	2.10E-74	18	met-d-Ph	rs9304381	0.818434	114999	1.70309	0.000844	1.591414	
52	Phosphati	-0.09847	0.00714	45413233	1.40E-44	19	met-d-Ph	rs1065853	0.080578	114999	2.421129	0.000245	0.461915	
53	Phosphati	0.024908	0.003883	49206985	2.10E-10	19	met-d-Ph	rs602662	0.534418	114999	1.316733	0.000178	0.335578	
54	Phosphati	-0.05082	0.006065	11190534	2.40E-17	19	met-d-Ph	rs1421589	0.116705	114999	2.056587	0.000126	0.237259	
55	Phosphati	-0.02939	0.005797	46402383	3.70E-08	19	met-d-Ph	rs8103923	0.129369	114999	1.965833	5.04E-05	0.094885	
56	Phosphati	-0.0448	0.004496	45444524	4.90E-24	19	met-d-Ph	rs7223511	0.705067	114999	1.524631	0.000359	0.67695	
57	Phosphati	-0.12348	0.010506	11347657	2.80E-32	19	met-d-Ph	rs737338	0.035186	114999	3.562607	8.16E-05	0.153697	
58	Phosphati	-0.08374	0.00738	19379549	6.70E-33	19	met-d-Ph	rs5854292	0.074383	114999	2.502713	0.000154	0.29053	
59	Phosphati	0.028179	0.00479	46340596	4.50E-09	20	met-d-Ph	rs4239651	0.794242	114999	1.624371	9.84E-05	0.185355	
60	Phosphati	-0.09188	0.011303	43042364	4.70E-16	20	met-d-Ph	rs1800961	0.030192	114999	3.833119	3.36E-05	0.063396	
61	Phosphati	-0.03648	0.005061	21916272	1.00E-13	22	met-d-Ph	rs5754102	0.183245	114999	1.716251	0.000135	0.254788	

Supplement Figure 1

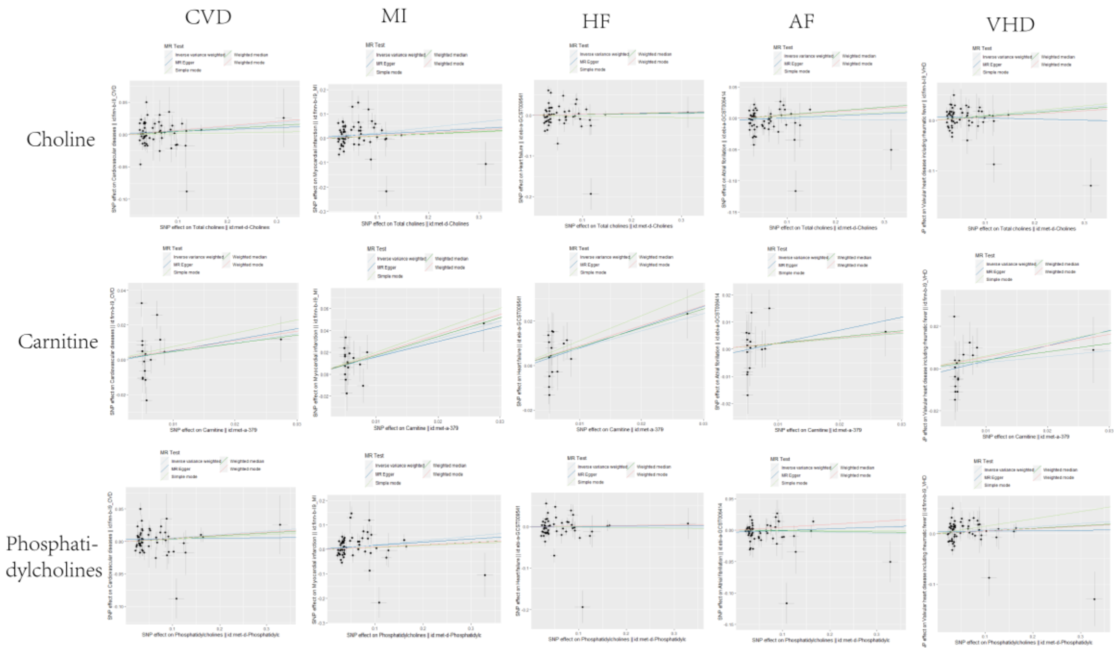

Supplemental Fig S1. The scatter plots of three dietary precursors for their effects (β values) on the risk of cardiovascular disease by SVMR. The horizontal axis represents the ability of SNPs to influence each dietary precursor, and the vertical axis represents the ability of each SNP to influence. Different lines represent the results from the specified analysis methods. CVD: cardiovascular disease; MI: myocardial infarction; HF: heart failure; AF: atrial fibrillation; VHD: valvular heart disease.

Supplement Figure 2

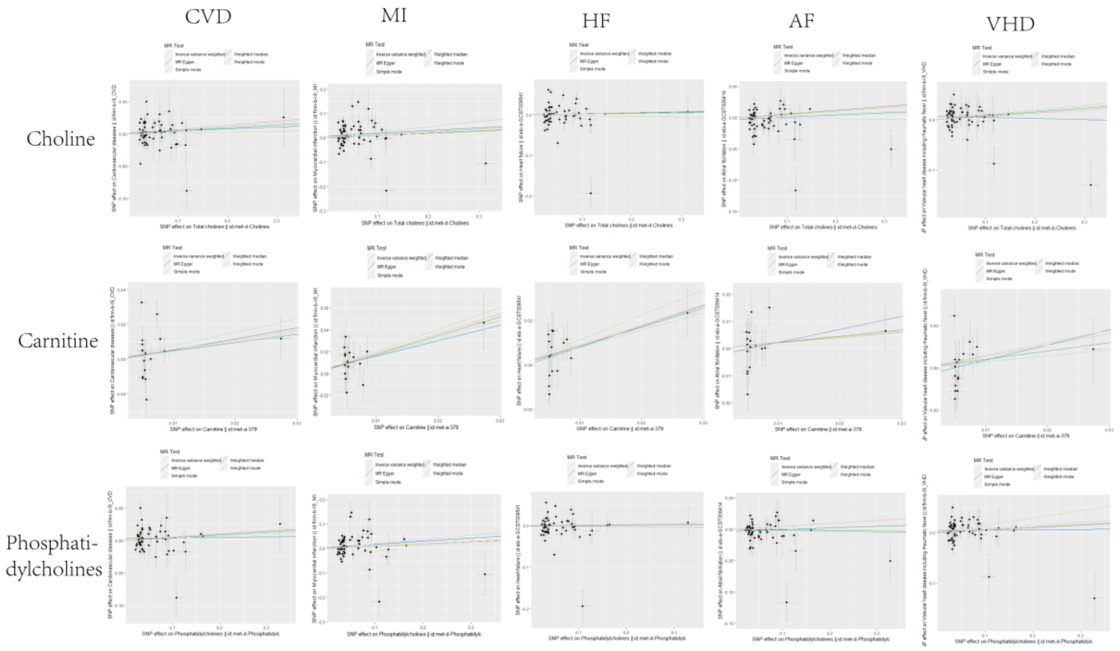

Supplemental Fig S2. The forest maps showing the effect of three dietary precursors on the risk of cardiovascular disease by SVMR analysis of SNPs. The abscissa represents the P value, and the ordinate is the ID number of each SNP. Line segments represent 95% confidence intervals for each SNP. CVD: cardiovascular disease; MI: myocardial infarction; HF: heart failure; AF: atrial fibrillation; VHD: valvular heart disease.

Supplement Figure 3

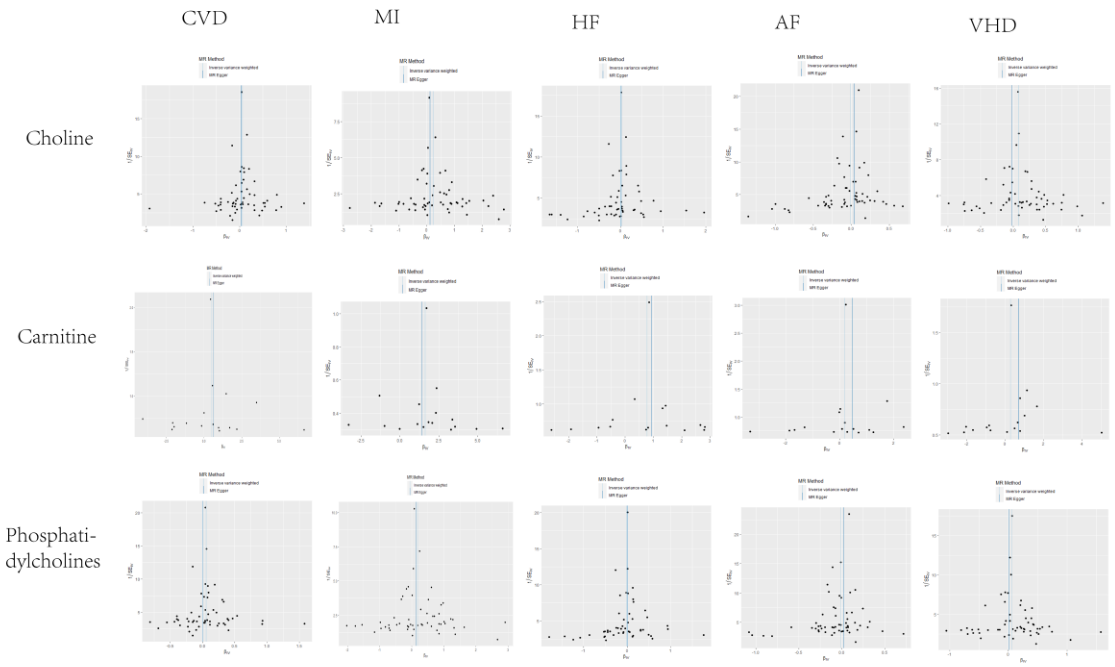

Supplemental Fig S3. The funnel plots of SVMR analysis of SNPs for the effect of three dietary precursors on cardiovascular disease by SVMR analysis. The abscissa represents the β value of each SNP, and the ordinate represents the reciprocal SE value. Dark blue lines represent the results of MR-Egger analysis and light blue line represent the results of IVW analysis. CVD: cardiovascular disease; MI: myocardial infarction; HF: heart failure; AF: atrial fibrillation; VHD: valvular heart disease.

Supplement Figure 4

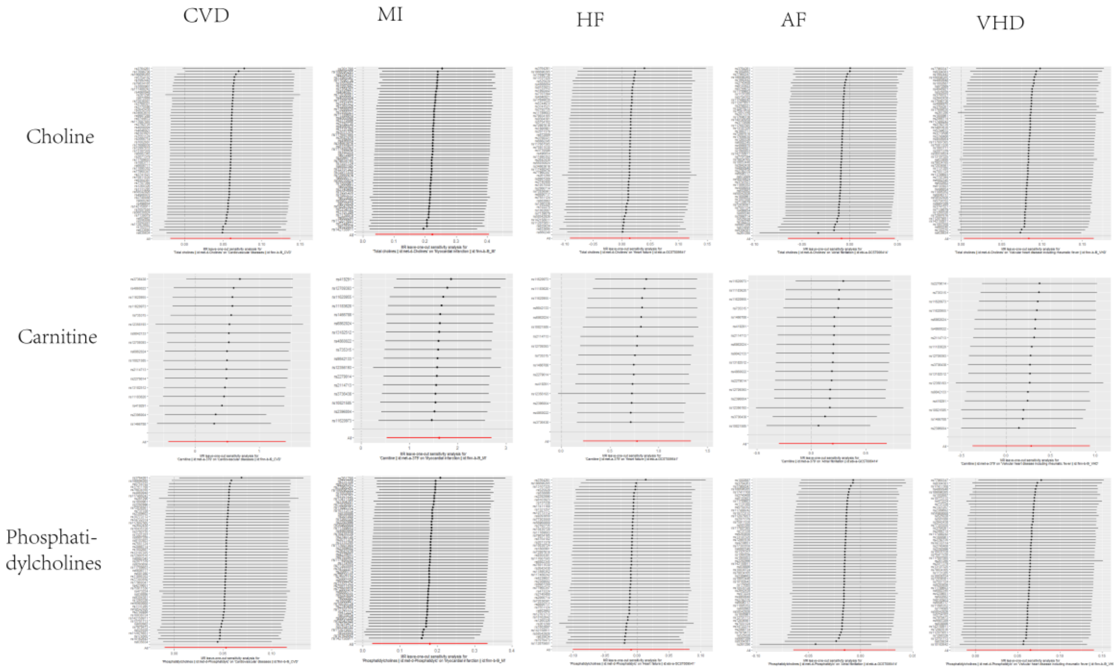

Supplemental Fig S4. The results of Leave-one-out sensitivity analyses. The abscissa represents the P value obtained by Leave-one-out method. The ordinate represents the ID number of each SNP. The red line shows the integration results of all SNPS. PC: phosphatidylcholine; CVD: cardiovascular disease; MI: myocardial infarction; HF: heart failure; AF: atrial fibrillation; VHD: valvular heart disease.
